# Case of Fracture of the Vertebra

**Published:** 1868-07-01

**Authors:** R. G. Bogue

**Affiliations:** Surgeon to Cook Co. Hospital, Chicago


### A CASE OF FRACTURE OF THE VERTEBRA.

BY R. G. BOGUE, M.D., SURGEON TO COOK CO. HOSPITAL, CHICAGO.

JOHN WIDDIS, aged 24, Irish laborer, was admitted into Cook County Hospital, April 14th, 1868. A few hours previous to admission he fell from a hand car while in motion, the car passing over him, quite doubling him up forward. Immediately after the injury, he noticed he had no control over the lower extremities, nor feeling in them.

On admission to hospital there was perfect consciousness, no pain, except at a point in the back, when the body was moved in any direction. At this point, which was in the region of the junction of the dorsal and lumbar vertebræ, there was moderate tumefaction, with tenderness on pressure, very little or no discoloration; a noticeable prominence of a spinous process, and near this, on either side, a hard body could be felt, supposed to be the transverse processes. There was no mobility of any of these prominent points. No other places of injury could be found. There was complete loss of sensation and motion of the lower extremities, the anæsthesia extending to a point about the hips, nearly as high as the cresta ilii. No urine passed since injury.

The diagnosis was: a fracture of the vertebra; and the patient put to bed, lying upon his back, there being apparently nothing to do for him except to minister to his wants in a general way as they should demand.

*April 17th.*—Patient about the same as when admitted; the bladder has been relieved four or five times a day with catheter; bowels not moved; a large injection has been used, but no response: three drops of *Croton oil* given, producing two copious evacuations; appetite fair; sleeps well; lies upon the back, and on either side, alternately; some pain at the point of injury whenever he is turned in bed. The prominent points, where pressure comes, begun soon to look a little red, as though they might slough.

*24th.*—Has remained much the same, except failed in strength and flesh; bed-sores have begun to form over the sacrum and trochanters; no pain; bowels moved by cathartics or enemata; urine drawn by catheter. There is now marked deformity at the point of injury, the spinous and transverse processes being plainly made out.

*May 9th.*—Has gradually failed, as above; bed sores rapidly becoming larger, and an extensive burrowing abscess has formed in one thigh and leg, discharging a large quantity of thin, offensive pus; sloughs have formed about the knees and ankles. The bed sores were dressed with *Permanganate of Potash* until the sloughs separated, then with *Carbolic acid.* Thin, fœcal matter leaks away from the bowels almost constantly; urine dribbles away; bladder not distended.

He has had one terrible paroxysm of pain, referred to the testicles, lasting an hour or so, relieved by large and repeated doses of *Morphine*, and warm fomentation to the parts. He was early put upon a water bed, but the bed sores continued to increase.

*May 14th.*—He is a good deal more prostrated and emaciated; a irritative or hectic fever has begun; pulse small, frequent and feeble, but little appetite, diarrhœa, sweats, sordes about the teeth, and low, muttering delirium. This condition continued until the morning of May 31st, when he died exhausted. He has had another severe paroxysm of pain in the testicles.

His mind remained perfectly clear until about one week before death, complained of no pain except the paroxysms above mentioned. His nourishment was liberal, and ale allowed. The temperature was noted repeatedly, being about two degrees higher below the injury than above it.

*Post-mortem* examination made about eight hours after death. The body very much emaciated, large and numerous bed sores upon the hips, and lower extremities; a marked deformity at the point of injury in the back. A section of the vertebral column was removed, revealing a fracture from below upward and backward of the body of the twelfth dorsal vertebra, and through the upper and outer part of the base of the transverse processes, and the ligamentous between the eleventh and twelfth dorsal spinous processes torn, the upper fragment, with the vertebræ above, shoved forward and downward, producing complete compression of the cord against the upper and posterior part of the lower fragment; the last ribs were partially torn from the vertebræ at their articulation, and twisted downward. The cord, about the point of compression was softened, the meninges adherent to the wall of the canal; in and about the injured parts there was a good deal of irrepairative material, a part of which had become bony; most of the inter-vertebral cartilage seems to have been destroyed by pressure of the fragments, or by absorption: a fragment of bone was separated and thrust upward between the vertebræ and the meninges.

